# Toxicity of Tire Rubber Microplastics to Freshwater Sediment Organisms

**DOI:** 10.1007/s00244-021-00905-4

**Published:** 2021-12-20

**Authors:** Victor Carrasco-Navarro, Aino Nuutinen, Jouni Sorvari, Jussi V. K. Kukkonen

**Affiliations:** 1 Department of Environmental and Biological Sciences, University of Eastern Finland, Kuopio campus, PO Box 1627, 70211 Kuopio, Finland; 2 Present Address: Natural Resources Institute Finland (Luke), P.O. Box 2, 00790 Helsinki, Finland

## Abstract

High emission of tire rubber particles to the surrounding environment is an inevitable consequence of the current habits of transportation. Although most of the emissions stay within a close range of the sources, it has been proven that the smallest particles can be transported to remote locations through the atmosphere, including inland water bodies. It has been estimated that a relevant portion of the global emissions of tire rubber particles reach surface waters, but effects on aquatic life in the receiving water bodies are not completely understood. In the present study, we used the freshwater sediment dwellers *Lumbriculus variegatus* and *Chironomus riparius* to examine the toxicity of tire rubber particles at environmentally relevant concentrations, using different types of sediment and two particle sizes of tire rubber. Overall, the experiments were unable to discern any effects on the growth, survival or reproduction of the two animals tested. Significant differences were found among the animals dwelling on different sediments, but the effects were not attributable to the presence of tire rubber particles. This study provides important information regarding the lack of effect of tire rubber particles in laboratory experiments with model sediment dwellers and opens more questions about the potential effects of tire rubber particles in the real environment with longer durations and varying environmental factors. The influence of other factors such as the leaching of additives in the overall toxicity of tire rubber particles should be also considered.

**Supplementary Information:**

The online version contains supplementary material available at 10.1007/s00244-021-00905-4.

Hydrogen- or electricity-powered vehicles are often touted as “low emission” or “no emission” mode of transport. However, seeing vehicle transportation as a whole non-exhaust emissions such as tire wear particles should be also considered (OECD 2020). Among the variety of plastics that we use in our society, synthetic rubber is one of the most widely used materials in appliances such as vehicle tires, hoses, footwear and rubber bands. By far, the production of vehicle tires is the dominant activity that uses synthetic and also natural rubber. The composition of tires consists of natural and synthetic rubbers (40–60%), fillers (20–35%), oils (15–20%) and vulcanization chemicals (4–5%), among others (Wik and Dave 2009). Although toxicity of whole tires has been considered (Day et al. 1993), the environmental impact of the particles generated during vehicle transit (tire wear particles; TWP) is the most common form of potential contamination for freshwater bodies (Kole et al. 2017). These TWP are generated through abrasion of the tires with the road surface. Due to the very high use of cars and the recommended substitution of their tires every thirty to forty thousand km, the emission of TWP to the environment is remarkable and estimated to be 6 million tonnes y^−1^ (Kole et al. 2017). Part of these emissions (about 0.1–10%) reach surface waters (Wagner et al. 2018), even in remote locations through atmospheric transportation (Evangeliou et al. 2020). However, little is known about the fate of TWP once they enter the aquatic environment. As a high percentage of the microrubber emissions to lotic water environments may be retained near the spots where it was originated (Besseling et al. 2017), we expect that a higher percentage of emissions to lentic freshwater bodies stay in the same lake or pond. Although information about the true levels of microrubber in the aquatic environment is scarce, it has been proven that sediments are a sink for plastic particles and their aggregates (Michels et al. 2018; Scherer et al. 2020). The rapid sinking of microplastics due to biofouling and aggregation with biogenic particles explains the unexpected low amount of microplastics in surface waters and underlines the importance of studies with microplastics sorbed to sediments.

As Halle et al. (2019) mentioned, there is a need to perform experiments on the toxicity of microrubber in different matrices and with varied organisms, as the reported information on microrubber toxicity is contradictory. Some of the chemical additives present in tire rubber are toxic to a wide variety of aquatic animals (Capolupo et al. 2020; Day et al., 1993; Gualteri et al. 2005), but the particles may also cause toxicity through physical damage of the gastrointestinal tract (GIT) after ingestion (Khan et al. 2019). Additives are chemicals that are added to the polymer to improve its durability and performance. For the additives to cause direct toxicity, it is necessary that they are taken up by organisms, either through leaching to water or to the GIT. Even if the leaching of the additives to the surrounding water is not relevant, it may be enhanced in the GIT due to digestive enzymes, like it has been suggested by Khan et al. (2019). This may increase bioaccumulation and toxicity of the additives, although the contribution of the plastics to the former may be very small if the concentrations of the additives in the environmental are high (Koelmans et al. 2016).

Physical damage or blockage of the GIT after ingestion is the main mechanism by which particles can threaten the health of an animal. The toxicity of microrubber depends on factors such as size and surface area of the particles, weathering, water physicochemical composition (pH, temperature), biofouling, sedimentation and particle ingestion.

*Lumbriculus variegatus* and *Chironomus riparius* are two widely distributed freshwater invertebrates used as model animals, as a first approach in chemical risk assessment and in bioaccumulation testing (OECD 2007 and 2010). They are easily cultured, offer several toxicity endpoints and are exposed to chemicals through various routes (Phipps et al. 1993). In addition, they are important members of the aquatic ecosystems, being prey of several predators.

The present study aims at studying the toxicity of microrubber to two representative freshwater sediment invertebrates, *L. variegatus* an *C. riparius*. The toxicity of two sizes of microrubber (fine and coarse sizes) at varying concentrations (ranging from 1 to 10% of sediment dry weight) and in sediments of different origin was tested by measuring growth and reproduction in both organisms and emergence in *C. riparius*.

## Materials and Methods

### Materials

Tire rubber was used in two particle sizes: fine, which size was characterized in Carrasco-Navarro et al. (2021), and large size particles, that were photographed with a Zeiss Axiocam ERc 5 s attached to an optical Zeiss Steni 508 microscope and measured with ImageJ. The origin of both types of particles was an undetermined pool of used tires at the end of their life cycle, ready to start the recycling processes, for example as crumb rubber in artificial turf sport fields. The fine-sized particles were provided to us already cryoground.

Representative samples of both sizes (11.3 mg of the fine-sized and 12 mg of the coarse-sized microrubber) were analysed by thermogravimetric analyses (TGA Q50 V6.7 Build 203, TA instruments) to quantify their contents following the procedure described in Redondo-Hasselerharm et al. (2018). Briefly, the samples were heated to 600 °C at a rate of 20 °C min^−1^ under N_2_ flow (50 ml min^−1^). Followingly, the gas carrier was switched to air (50 ml min^−1^) and the heating continued until 850 °C (20 °C min^−1^).

### Test Organisms

*Chironomus riparius* (Diptera: Chironomidae) were reared in aquaria covered with nets and consisted of sieved (1 mm) lake Höytiäinen sediment (62° 41′ 21′′ N 29° 40′ 34′′ E) and artificial freshwater (AFW; hardness Ca^2+^ and Mg^2+^ 0.5 mM, pH = 6–9) at 20 ± 1ºC and 16:8 h light–dark period. Larvae were fed powdered Tetramin® fish food (Tetrawerke, Germany) three times a week, and overlying water was renewed once a week.

Larvae used in all the experiments were obtained by placing adults in a rearing cage containing a beaker with aerated AFW, where egg ropes were planted. Egg ropes were monitored for hatching and all the experiments were initiated when the larvae were 1–3 d old. On the water surface, chironomids may lay hundreds of individual eggs organized in gelatinous masses called egg ropes.

*Lumbriculus variegatus* (Oligochaeta) were reared in aquaria similar to those used for *C. riparius* but containing AFW 1 mM in Ca^2+^ and Mg^2+^ (renewed once a week). Dechlorinated paper towels were used as substrate, and Tetramin was added as food source 3 times per week.

In both organisms, the aquaria were commercially available with variable sizes (L 30–40 cm, H 12–40 cm, W 18–26 cm). In *C. riparius*, the nets were handmade with heights ranging from 37 to 44 cm.

### Sediments

Three sediments were used based on availability: lake Höytiäinen (62°41′ 32.5" N 29°40′ 22.6" E, collected in June 2019 by a suction pump and wet-sieved (1 mm) on site), lake Ruosmanlampi (62°56′16.5" N 30°03′03.3 "E, collected in November 2017 by Ekman sampler) and Huruslahti bay (62°19′ 09.3"N 27°52′ 51.5" E, collected in October 2012 by Ekman sampler). If sediments were not sieved on site, they were wet-sieved (1 mm) in the laboratory, frozen at − 20 °C for 48 h and stored in the dark at 4–6 °C until the beginning of the tests. Sediments dry weight was calculated by weighing the sediment residue after sediment samples were dried overnight at 105 °C (n = 3 for each sediment).

In all cases, microrubber was spiked in the sediment following van Weert et al. (2019) with minor modifications. Briefly, sediment was added to a glass beaker and the needed quantities of microrubber were added and mixed manually with a metal spatula. Followingly, beakers were introduced in a water bath and sonicated for approximately 12 min, while they were further mixed with a domestic electric mixer. Microrubber was not rinsed before spiking. The concentrations of microrubber were 1, 3 and 10% of sediment dry weight in the experiments with *C. riparius* and 1, 5 and 10% sed dwt in the experiments with *L. variegatus*. These concentrations are in the range of concentrations found in the environment that are up to 15.5% (Wik and Dave, 2009).

The nominal concentrations of the fine microrubber in the Höytiäinen sediment were evaluated by measuring the extractable quantities of zinc in sediment samples with nominal concentrations of microrubber of 1, 3, 5 and 10% of sediment dwt. Samples (*n* = 3) were extracted by sonication with 20 ml of acetic acid 0.5 M (Trace metal grade, Fisher Scientific) in 50-ml glass test tubes for 20 h. Control, unspiked sediments and a blank were also set to calculate the background concentrations of zinc, and the values obtained in the sediments were corrected for these values. As sediments tend to precipitate in the bottom on the tubes despite the sonication, sediments were suspended in the acid solution when possible.

In order to determine the extractable concentrations of Zn on microrubber, samples of different weights (8.7 ± 2.5 and 38.4 ± 2.2 mg, *n* = 3) were extracted similarly as the sediment samples. The total Zn extracted was plotted against the weight of microrubber (mg) extracted. To complete a more accurate regression line (Fig. S1), the samples extracted in our previous work (Carrasco-Navarro et al., 2021; 93.6 ± 6.8 mg) were also considered despite a slightly different extraction method. The linear regression obtained from the extraction of microrubber was used to estimate the weight of microrubber present in the extracted sediments by extrapolation of the Zn concentrations obtained.

All the samples were filtered through MCE membranes (pore size 0.22 µm), and the resultant acidic waters were run in ICP-MS as described in Carrasco-Navarro et al. (2021). To evaluate the measured concentrations of the sediments containing the coarse microrubber, we sieved (100µm) the used sediment and collected the rubber particles found. They were cleaned with 30% H_2_O_2_ at 45 °C overnight and weighed. It was reported that this treatment does not digest tire rubber at all (Redondo-Hasselerharm et al. 2018).

### Experiments with Chironomus Riparius

In all experiments with *C. riparius*, adults from our permanent cultures were allowed to lay egg ropes in a glass beaker. The egg ropes were monitored, and once larvae hatched, they were fed Tetramin. As both egg laying and larval hatching occur gradually over several days, larvae used in the toxicity tests were 1–3 d post-hatching. For the growth and emergence tests, 30 and 15 egg ropes were used as a source of larvae, respectively.

#### Growth Tests

The experimental vessels consisted of glass flasks (⁓ 5.5 cm in diameter) containing 20 g of lake Höytiäinen sediment at concentrations of 0 (controls), 1, 3 and 10% of fine or coarse microrubber (seven treatments), 100 ml of AFW 0.5 mM and eleven larvae. Tetramin was added at a rate of 0.36 mg larvae^−1^ d^−1^ every other day, and aeration was set after approximately 24 h to facilitate larvae to settle in the sediment. Experimental waters (50 ml) were renewed every 4 d. Temperature was 20 ± 1 °C.

After approximately 10 d of exposure, alive larvae were sampled, counted and stored in ethanol at − 20 °C until further measurements of body length and head capsule lengths and widths.

#### Emergence Tests

Sediments from lakes Höytiäinen and Ruosmanlampi were used in the emergence experiments. The experiments with sediment from Ruosmanlampi included the fine-sized tire rubber at concentrations of 1, 3 and 10% of sediment dry weight plus control treatments (all *n* = 4). Experimental vessels consisted of 600-ml beakers filled with 30 g of sediment and 250 ml of AFW. The experiment was considered as started when twenty-five first instar, 1–3 d old larvae were added to the prepared experimental vessels. Larvae were fed Tetramin every other day at a rate of 0.36 mg larvae^−1^ d^−1^ and approximately 24 h after the introduction of the larvae, aeration was set and maintained until the end of the tests. A light/dark cycle of 16:8 h was used, and temperature was 20 ± 1 °C. On d 2, the water pH was 4.5, and following the pH recommendations for chironomid sediment tests (pH = 6–9), 80% of experimental waters were changed on d4 to a 0.5 mM AFW that included a phosphate buffer. The measured pH after the substitution of the waters stayed well between the recommended values. The same water change procedure was repeated every 3 or 4 days (d8, 11, 14, 17, 20, 23 and 26).

As the results with the Ruosmanlampi sediment did not offer a definitive conclusion about the toxicity of microrubber, we conducted additional experiments using a reference sediment, used previously in several studies (e.g. Leppänen and Kukkonen 1998). The experiments with lake Höytiäinen included two sizes of tire rubber (fine and coarse), both at the same concentrations (1, 3 and 10% of sediment dry weight). Control replicates were also set, using sediment without microrubber. Experimental vessels consisted of 600-ml glass beakers filled with 50 g of sediment, 250 ml of AFW made as described above. Four replicates per treatment were set (24 exposed and 4 controls), and 25 larvae were added per replicate (total of 600 exposed larvae and 100 control larvae). In this occasion, the pH was within the recommended limits, so a buffer was not needed. Experimental waters (80%) were changed every four days, and light/dark cycle and food rate were the same as in the previous experiment. Temperature was 21 ± 1 °C.

In both tests, experimental vessels were covered with mosquito nets to avoid the escaping of adults. Emergence of males and females was monitored every day, and they were transferred to reproduction cages corresponding to their treatments. The emergence of males and females separately was also investigated for significances compared to the controls.

#### Reproduction

In both emergence experiments with Ruosmanlampi and Höytiäinen sediments, adults emerged in every replicate were transferred daily to a reproduction cage established for every treatment (total of four cages for experiments with Ruosmanlampi sediment and seven cages, for experiments with Höytiäinen sediment). Reproduction cages (59 × 33 × 33.5 cm) were made of methacrylate and nets, with two apertures that facilitated the insertion of the adults. The individual beakers with visible emerged adults were carefully introduced in the cages, and the covering nets were removed to let adults free inside the cage. Once the adults were in the reproduction cage, the beaker and its net were taken out from the cages and set as before to receive more emerging adults the following days. Clean AFW was made available in two petri dishes inside the reproduction cages for adults to lay egg ropes. Egg ropes were counted daily, and the number of egg ropes per female was calculated after 28 d.

### Experiments with Lumbriculus Variegatus: 28d Growth and Reproduction Tests

Sediment from lakes Höytiäinen and Huruslahti, prepared as described above, was used as substrate in these experiments. One week before the start of the tests, the reproduction of *L. variegatus* was synchronized by cutting individuals longer than 4–5 cm in two parts with a blade.

Experimental vessels consisted of 400 ml glass beakers filled with 50 g of fresh sediment and 200 ml of buffered AFW 1 mM. As a preliminary test revealed that the pH of Huruslahti bay sediment decreased below the recommended values (6–9), it was decided to use a phosphate buffered AFW 1 mM, made similarly as in the experiments with chironomids.

Twelve worms were added per replicate and aeration was set after 24 h. Light/dark cycle was 16:8 h, and worms were not fed during the tests. The beakers were covered with parafilm to minimize the evaporation of the experimental waters. Approximately 150 ml of experimental waters was changed once a week and water parameters (pH, conductivity and dissolved oxygen) measured. After 28 d, worms were sampled from sediment, counted and transferred to AFW for 6 h to empty their guts. Followingly, worms were placed in previously weighed aluminium foil cups, dried overnight at 105 °C and weighed.

### Statistical Analyses

All the experimental variables were tested for statistical differences with one- or two-way ANOVA (Sigma Plot v14). Normality and equal variances of data were tested with Shapiro–Wilk and Brown–Forsythe tests. In the case that the data did not meet the normality test, a Kruskal–Wallis one-way ANOVA on ranks was performed. The cumulative emergence % of larvae (also of males and females) was tested for significant differences with a log rank survival test (Sigma Plot v. 14). In case of significant differences, pairwise tests applied were Holm-Sidak or Dunnett. Differences were considered as significant if *p* ≤ *0.05*.

## Results

### Material Characterization

As reported in Carrasco-Navarro et al. (2021), the sizes of the fine microrubber were 82.3 ± 40 μm and the measurements from the coarse microrubber gave a size of 3724 ± 775 μm (Fig. S3).

The TGA analyses (Fig. S4 and Table S1) revealed a content of volatile compounds of ⁓ 6% (heating between 30 and 300 °C), 54–58% of polymers (determined by loss of weight between 300 and 600 °C), 31–33% content of black carbon (600–850 °C) and 5–7% of residue that comprises inorganic fillers.

### Water Parameters

Water parameters (pH, conductivity and dissolved oxygen) were measured in the *L. variegatus* experiment weekly. The water pH was measured in three random samples 3 times per week and was almost identical in both Huruslahti and Höytiäinen treatments (6.75 and 6.76, respectively). One value on Huruslahti treatment was slightly lower than the recommended values (5.5). Average conductivity in Huruslahti was 329 and in Höytiäinen 351 μS cm^−1^. Oxygen levels were over 92% of saturation in all cases.

In the *C. riparius* experiments, O_2_ was measured from the water column in the Ruosmanlampi treatment in four replicates at the end of the test (over 80% of saturation), conductivity was measured in the lake Höytiäinen treatment in five replicates (153–202 μS cm^−1^) and pH was measured in Ruosmanlampi treatment during the first 4 d (4.56–5.05) and after the water was changed for a phosphate buffer (6.3–7.3). The pH in the water column from the Höytiäinen sediment treatment was measured the first 5 days (6.7–7.3). These values were the base to decide the water change every 5 days. Temperature was 20 ± 1 °C in all cases.

### Measured Concentrations of Microrubber in Sediment

The measured concentrations of the fine microrubber in spiked Höytiäinen sediments (average ± SD) were 8 ± 2.1, 24.33 ± 1.9, 29.5 ± 0.04 and 75 ± 6.4 mg g sediment dwt^−1^ (Table 1). These correspond to the treatments with nominal concentrations of 1, 3, 5 and 10% of microrubber sediment dry wt^−1^. Regarding the coarse microrubber, used in the *C. riparius* growth and emergence experiments, the measured concentrations were 13.1 ± 2.3, 30.6 ± 2.3 and 115 ± 20 mg of microrubber g of sediment dry weight^−1^. These correspond to the 1, 3 and 10% of sediment dry weight nominal concentrations and are more accurate than the values obtained from the sediments with fine microrubber (Table 1).

**Table 1:** Emergence experiment, treatments, subtreatments and parameters measured

Sediment	Treatment	Measured % of rubber in sed. dwt ^−1^	E_R_	Sex ratio	T for 50% E_R_	Eggs ropes/♀
Höytiäinen	Control	–	77.3^*^	1.03 (0.3)	15 (0.25)	0.7
	Coarse 1%	1.3 (0.23)	69	1.3 (0.46)	15.2 (0.26)	0.65
	Coarse 3%	3.06 (0.23)	82	1.3 (0.9)	15.3 (0.22)	0.74
	Coarse 10%	11.5 (2)	71	0.95 (0.4)	14.8 (0.23)	0.66
	Fine 1%	0.8 (0.2)	66	0.82 (0.1)	15.01 (0.3)	0.76
	Fine 3%	2.43 (2)	70	1.33 (0.96)	14.8 (0.18)	0.56
	Fine 10%	7.5 (0.6)	71	1.33 (0.99)	15.14 (0.26)	1.15
Ruosmanlampi	Control	–	85	1.4 (0.44)	20.02 (0.33)	0.42
	Fine 1%	N.d	74	1.05 (0.14)	17.6 (0.3)	0.33
	Fine 3%	N.d	91	0.99 (0.06)	17.6 (0.23)	0.52
	Fine 10%	N.d	80	1.05 (0.56)	18.11 (0.3)	0.5

Measured % concentration of microrubber in sediment dry weight (sed. dwt). E_R_: emergence rate % (28d), T = time. Egg ropes per female are shown in the last column. In parenthesis is shown the standard error. * Is the emergence rate without the outlier control beaker

### Tests with Chironomus riparius

#### 10d growth Tests

**Fig. 1:**
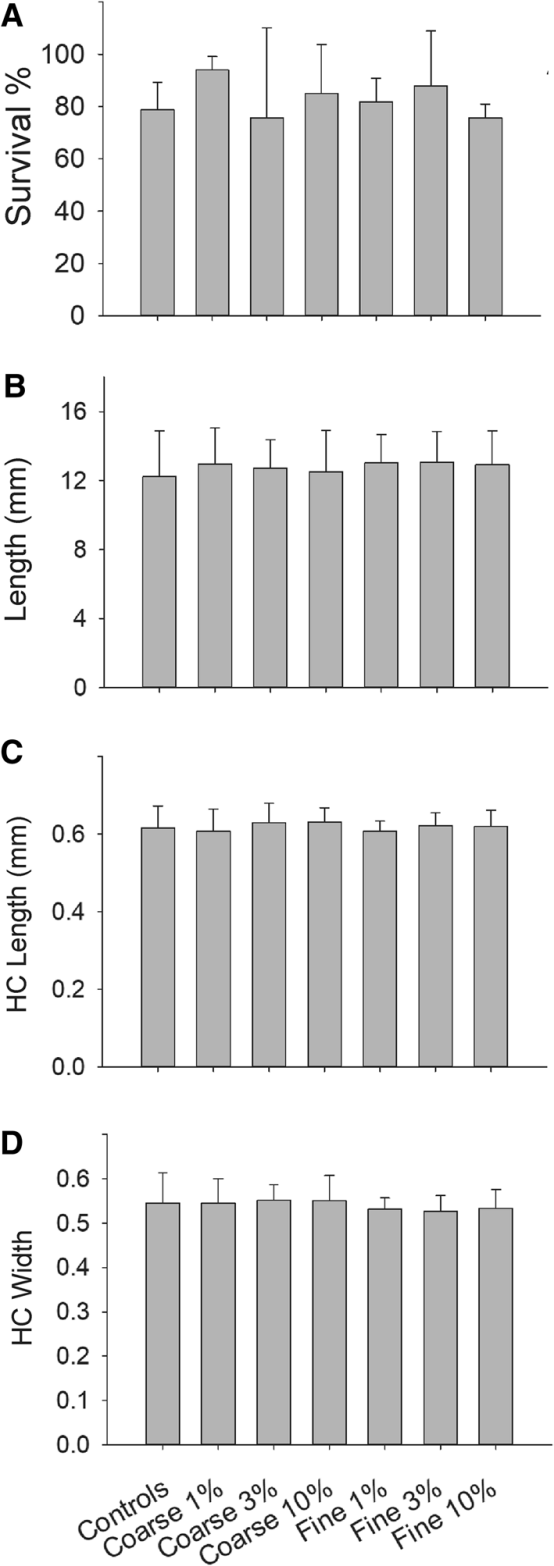
Growth of *Chironomus riparius* larvae in Lake Höytiäinen sediment. In **A,** it is shown the survival % of the larvae, in **B** the total length of the larvae (mm), in **C** the head capsule (HC) length and in **D** the head capsule width. From left to right bars show the treatments controls, coarse-sized microrubber 1, 3 and 10% nominal concentrations, Fine-sized microrubber 1, 3 and 10%

The endpoints measured during the growth tests are shown in Fig. 1A–D. The survival of larvae exposed to microrubber was not affected in any of the concentrations (Fig. 1A; two-way ANOVA, F = 0.273, df = 2, *p* = 0.766) or sizes tested (Two Way ANOVA, F = 0.117, df = 1, *p* = 0.738). The survival of the control larvae was in average 78.8%, and over 70% in all the replicates, fitting the conditions stated in the OECD guidelines (OECD 2010).

The length of the larvae did not depend on the concentration of microrubber present in the sediment (Fig. 1B; one-way ANOVA, H = 1.559, df = 9, *p* = 0.955). The same conclusions were obtained for the head capsule length and width (Fig. 1C, and D). Although the Kruskal–Wallis ANOVA indicated a difference among the head capsule widths in the different treatments, pairwise comparisons did not find any.

#### Emergence Tests

**Fig. 2:**
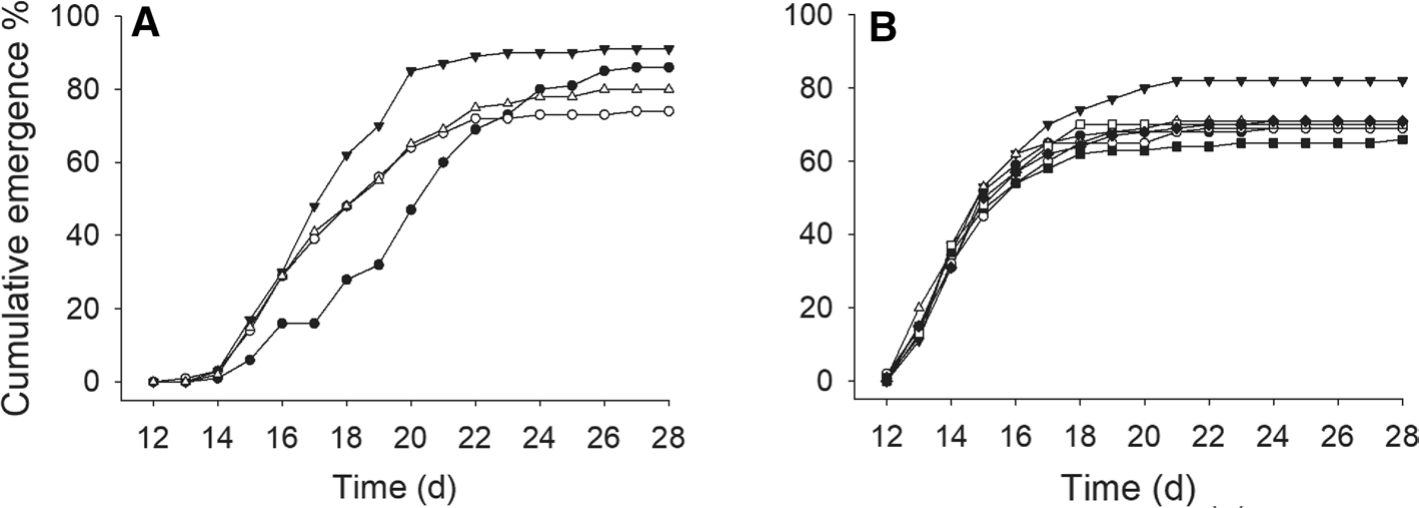
Emergence profiles of C. riparius in lake Ruosmanlampi sediment (**A**) and lake Höytiäinen sediment (**B**). In A, black circles represent sediment control, white circles concentration 1%, inverted triangle concentration 3% and triangle concentration 10%. In **B**, black circles represent sediment control, white circles coarse-sized, concentration 1%, inverted black triangle coarse-sized, concentration 3%, triangle coarse-sized, concentration 10%, black square is fine-sized, 1% concentration, white square is fine-sized, 3% concentration and diamond fine-sized, 10% concentrations

The cumulative emergence profiles of *C. riparius* in both sediments are shown in Fig. 2A and B. In sediments from Ruosmanlampi, larvae in control sediment did show a remarkably different cumulative percentage compared to the exposed larvae (Fig. 2A), which was confirmed during the statistical analyses (log rank survival test, statistic = 35.528, df = 3, *p* < *0.001*). However, the final emergence rates (E_R_, defined as the number of emerged adults divided by the number of introduced larvae) on d 28 did not differ (Kruskal–Wallis One Way ANOVA, H = 0.625, df = 3, *p* = 0.891). To see whether these results occur in other sediments, we tested the same endpoints in sediment from lake Höytiäinen, adding one extra size of microrubber to the experimental setup. The cumulative emergence was not different in any of the concentrations or sizes tested (log rank survival test, statistic = 1.381, df = 6, *p* = *0.270*). The E_R_ on d 28 was also similar among the treatments (one-way ANOVA, F = 0.869, df = 6, *p* = *0.534*). Considering both tests as a whole, microrubber spiked to two sediments, in two sizes and three concentrations did not cause any alterations in the emergence of the larvae compared to their respective controls. Regarding the validity of the test, the 28 d emergence rates for controls in sediments from lakes Ruosmanlampi and Höytiäinen were 85 and 69%, respectively. One replicate in each sediment did not comply with the OECD guidelines, in the case of Ruosmanlampi, it was 68% and in the case of Höytiäinen, it was 44%. We accepted the value in the Ruosmanlampi sediment because it was just one animal away from acceptable rate and did not interfere with the overall results. In the case of lake Höytiäinen, we performed analyses with and without this value, with no changes in the conclusions, so it is our belief that something occurred with this replicate and therefore we discarded its value. The 28 d E_R_ for controls in lake Höytiäinen without this value was 77.3%. Overall E_R_ for all sediments and concentrations is shown in Table 1.

##### Emergence of Males and Females

The emergence profile of males and females is shown in Fig. S2. In the Ruosmanlampi sediment, the emergence of both males and females in all the microrubber experiments differed from the emergence of control larvae (survival log rank test, *p* < *0.001*). However, in the Höytiäinen sediment, the emergence profiles were not different (males *p* = *0.270*; females *p* = *0.602*). These results indicated the lack of effects of microrubber to female or male emergences.

#### Reproduction Tests

The number of egg ropes produced per female is shown in Table 1. In general, the females produced the same number of egg ropes despite the concentrations of microrubber that they were exposed to, except in the treatment with highest microrubber concentration of 10%, at the small particle size, where an increase of the production egg ropes was observed (1.15 egg ropes female^−1^). However, each egg rope contains a variable number of eggs, so we cannot reach a solid conclusion about this fact. In addition, we cannot compare the results statistically, as we transferred all the adults from each replicate (*n* = 4) to the same reproduction cage; therefore, we only have one replicate of reproduction.

### Tests with Lumbriculus Variegatus

**Fig. 3:**
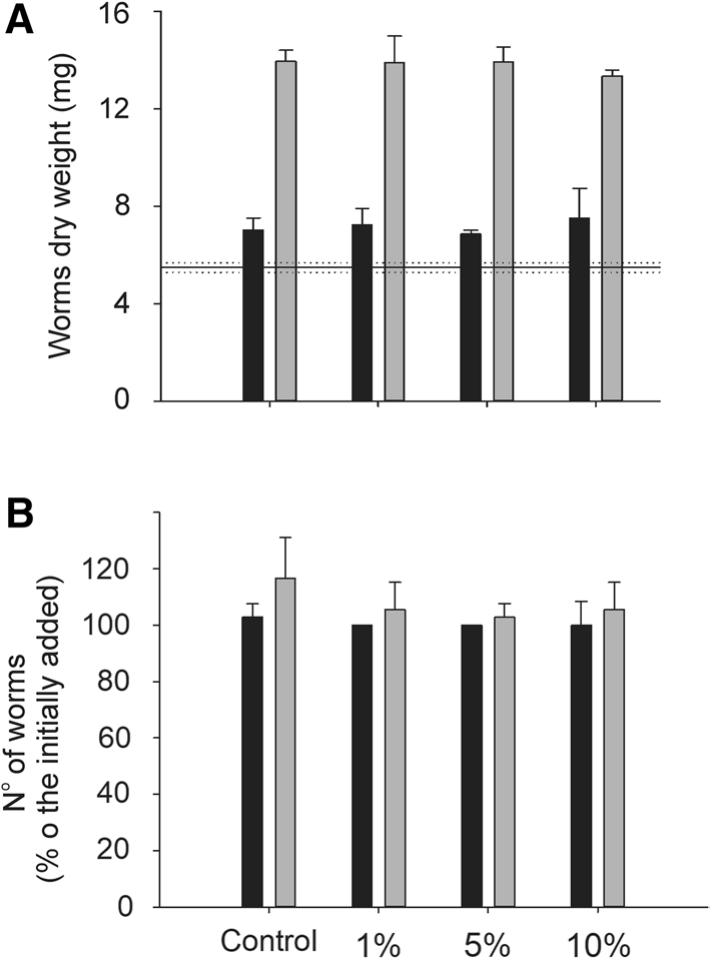
Experiments with *Lumbriculus variegatus* in lakes Höytiäinen (black bars) and Huruslahti (grey bars) sediments. In **A**, the dry weight of worms (mg) is represented. The horizontal lines represent the average dry weight of 12 worms when the test started (dotted lines show the standard deviation). In **B**, the reproduction of the worms is shown. The number or worms added at the beginning of the tests was considered as 100%, and the % of worms at the end of the test is shown based on it

The addition of fine microrubber at concentrations of 1, 5 and 10% of sediment dry weight did not alter the reproduction (Kruskal–Wallis one-way ANOVA on ranks, H = 8.576, df = 8, *p* = 0.379) or growth (two-way ANOVA, F = 0.0682, DF = 3, *p* = 0.976) of *L. variegatus* in any of the two sediments used (Fig. 3A and B, respectively). However, there was a difference in the growth (dry weight) between worms in the two sediments (two-way ANOVA, F = 515.307, df = 1, *p* < 0.001). These differences were also evident while comparing the dry weights within the concentrations used (Fig. 3B).

## Discussion

The present article did not find any effect caused by tire rubber to the sediment dwellers *L. variegatus* and *C. riparius* in laboratory experiments, which is in agreement with the literature published on the subject (Redondo-Hasselerharm et al. 2018) Although the findings were consistent despite the different factors used in the experimental design, we recognize the need for additional studies with species collected in the environment, longer exposure times and taking into account other potentially hazardous factors such as the leaching of additives.

### Characterization of the Materials

The content of microrubber in volatile compounds (⁓ 6%) and polymers (54–58%) is in accordance with analyses performed previously (Redondo-Hasselerharm et al., 2018). However, the black carbon content in our samples is approximately 5 times higher than in that study, while the residue was 5–7% in our study and five times higher in the study by Redondo-Hasselerharm et al (2018). However, the percentages of carbon black and residue in crumb rubber from scrap tires reported by Wang et al. (2020) were similar to our results, which suggests their validity. The clear differences in the contents of carbon black and residues might be due to the origin of the particles used (tread tire particles vs particles from whole tire).

### Concentrations of Tire Rubber in Sediments

The concentrations used in the present study are within the concentrations of microrubber found in the environment (reviewed in Baensch-Baltruschat et al., 2020), with a maximum of 15.5% of sediment dry weight (Spies et al., 1987). The method that we used to quantify the presence of tire rubber in sediments is based on the high content of Zn in tire rubber (Councell et al. 2004). In general, the calculated values of the fine-sized microrubber in the sediment are underestimated compared to the nominal concentrations (Table 1). While acetic acid 0.5 M may be a good method to extract the zinc present in tire rubber (Canepari et al. 2018), it may not be a completely suitable method to determine the real content of microrubber in sediments. As the particles may be covered and interacting closely with sediment particles and microorganisms, the extraction effectivity of zinc possibly decreased, causing the observed underestimation of the nominal concentrations. The lowest extracted mass of microrubber (8.7 ± 2.5 mg) was spiked in sediment and extracted, yielding higher quantities of zinc per microrubber mass unit than in our previous study (Carrasco-Navarro et al., 2021). A possible justification is that longer extraction times and lower mass of rubber extracted (despite being in sediment) yield higher concentrations of zinc extracted. It is also possible that the effectivity of Zn extraction would decrease with increasing tire rubber concentrations in sediment. An alternative and more efficient method is to digest sediments completely with a concentrated acid (van Griethuysen et al. 2004; Redondo-Hasselerharm et al. 2018). This method would also digest the present microrubber particles and dissolve the present amount of zinc. Our method only partially dissolves the present zinc, but its increasing concentrations in the spiked sediments prove that the spiking method was successful, as demonstrated by Redondo-Hasselerharm et al. (2018).

As zinc has been determined to be the best marker for the quantification of tire rubber particles (Klöckner et al. 2019), it could be argued that its total concentration in sediments could be used to determine the contamination of tire rubber, but a pristine sediment would be needed as a reference. More recent methods involving the use of pyrolysis GC–MS and the identification of specific synthetic rubber chemicals such as vinylcyclohexene and cyclohexenylbenzene could be also used (Goβmann et al. 2021), although this equipment is not as widely distributed and easy to use as other digestion appliances. Additionally, N-cyclohexyl-2-benzothiazolamine (NCBA), a benzothiazole derivative, has also been identified through GC–MS as a marker of tire rubber contamination (Knight et al. 2020).

Regarding the coarse-sized microrubber, their measured concentrations (Table 1) were similar to the nominal concentrations used, indicating a good distribution of the particles within the sediment with the spiking method that we used.

### Toxicity of tire Rubber to Lumbriculus Variegatus and Chironomus Riparius

The lack of toxicity underlined in the present study is in agreement with other studies that reported similar findings (Marwood et al. 2011), even using *L. variegatus* or a similar chironomid species as model animals (Redondo-Hasselerharm et al. 2018 and Panko et al. 2013, respectively). As sediment is the main sink for most types of microplastics (Scherer et al. 2020), including tire rubber (Marwood et al. 2011), studies that explore their behaviour and toxicity in this environmental matrix are a priority. Similar to what has been observed with other microplastic particles, it is likely that the microrubber particles form homo- and heteroaggregates with other biogenic and inorganic particles and, depending on the density of the particles and of the final aggregate, increase their sinking rates (Brewer et al. 2021; Michels et al. 2018). These processes are enhanced if the microplastics have been previously covered by biofilm. Although the formation of aggregates and their subsequent sedimentation may be very rapid, the mechanisms that lead to the permanent accumulation of microrubber particles in sediments are partially unknown and may depend on bioturbation, turbulence and overall sedimentation rates. The distribution of microplastics in the upper layers of sediment reflect the anthropogenic activity and sedimentation patterns (Zheng et al. 2020) and shows that the sedimentation of microplastics takes decades. It can be considered that the concentrations we used are similar to the concentrations found in environmental sediments (Baensch-Baltruschat et al., 2020) that have been reported in the range of non-detected to a maximum of 155 mg tire rubber g sediment dwt^−1^ (15.5% of sediment dry weight). In addition, the sonication of the sediments spiked with microrubber would facilitate the formation of heteroaggregates (van Weert et al. 2019), mimicking and accelerating the reproduction of environmental conditions.

The present study intended to be as environmentally relevant as possible, and it succeeded in some aspects and failed in others. Although we did not find a negative effect of microrubber considering several important factors (size, sediments, different animals), this does not mean that microrubber is a harmless material. Clearly, more ecologically relevant experiments including in situ, mesocosms, longer duration and more variable conditions should be performed to confirm the findings reported in this study. Variations in the temperature, sediment organic carbon, oxygen saturation, pH, hardness and organic matter are only few of the factors that may influence the toxicity and that were not completely taken into account in the present article. Also, the chemicals present in tire rubber need to be the focus of the research efforts when talking about ecotoxicity effects, as it has been the case in the last years (Capolupo et al., 2020; Tian et al., 2021).

Other studies have found similar results with several species of aquatic animals using sediment as source of the microrubber particles or mimicking environmental conditions (Marwood et al. 2011; Panko et al. 2013; Redondo-Hasselerharm et al. 2018), while other authors that used microrubber particles suspended in water or tire rubber leachates found more complex and adverse effects (Carrasco-Navarro et al. 2021; Gualteri et al. 2005; Halsband et al. 2020; Khan et al. 2019; Stephensen et al. 2003; Wik et al. 2009). In our study, the possible toxic effects of the leaching additives are masked by the frequent change of overlying waters that would dilute the potentially leached chemicals. Some authors compared the toxicity of the particles and the leachates (Halle et al. 2021), finding that in general the particle suspension was more toxic than the leachate. These results and our study point out that the differences in the matrix used as a source of the particles or additives seems to be crucial for the observed toxicity. The presence of natural particles reduces the ingestion and intensifies the egestion of microplastics (Scherer et al. 2017). Therefore, as sediments are rich in natural particles, the ingestion and retention of microplastics is lower than in water exposures, which is likely occurring for microrubber as well. For example, at 10% concentration of microrubber of sediment dry wt, a very low number of tire rubber particles were ingested by *Gammarus pulex* (Redondo-Hasselerharm et al. 2018), while *Hyalella azteca* exposed in water was found to ingest the particles “indiscriminately” (Khan et al. 2019). In addition, feeding behaviour and the size of both the organism and the microplastics also influence their ingestion (Scherer et al. 2017 and 2018). In our study, *Chironomus riparius* was exposed to two sizes of microrubber in three concentrations and two durations (10 d and whole larval stages), with no effects found. Assuming that the toxicity originates with the ingestion of particles, it is obvious that the coarse-sized microrubber would not cause any threat, as the results confirmed in both the growth and emergence tests. However, at least a part of the fine-sized microrubber (average and minimum size 82.3 and 2.45 µm, respectively) could theoretically be ingested and cause gut damage, although only during a limited time frame. As the *C. riparius* larvae were added as first instar (mean head capsule width of 120 µm, Watts and Pascoe 2000), this reduces the size of the particles that they can ingest. Although the possible sizes of microplastics physically possible to ingest would increase with the growth of the larvae and its head capsule, the limit is dictated by the mentum, at approximately 100 µm width (Scherer et al. 2017). Therefore, approximately 50% of our fine-sized microrubber would be too large for *C. riparius* to ingest. This limit, together with the apparent low ingestion and high egestion rates, reduces the potential toxicity to *C. riparius* in long sediment tests. In previous studies, we have found changes in the gene expression of the same organisms in response to the same fine-sized microrubber (Carrasco-Navarro et al. 2021). The likely cause was the oxidative stress caused by ingestion of the particles. Although the concentrations and experimental time used were low, exposures were performed in water and fourth instar larvae were used, which increases the chances for ingestion. In the future, more studies examining the effects at the molecular level with longer exposure times in sediment need to be performed.

In the case of *L. variegatus*, the addition of the fine-sized microrubber at the same concentrations as in the experiments with *C. riparius* did not cause any toxic effects. *Lumbriculus variegatus* feeds actively below the sediment surface, while *C. riparius* feeds at the sediment surface. Therefore, *L. variegatus* had a higher probability of ingesting microrubber, but still no toxicity was found. In fact, after the 6 h used to egest their gut content, microrubber particles were observed in the faeces in the worms exposed to the highest concentration, although they were not quantified.

Another factor that may influence the toxicity is the age, being the microrubber originated from pristine tires more toxic than the worn tires (Halle et al. 2021). Our microrubber particles originated from tires at the end of their life cycle, so it may be possible that particle coming from pristine tires would cause toxicity, although its environmental relevancy needs to be proven.

Regarding the two sediments used, the intention was to test whether existing sediment contamination would influence the potential toxicity of microrubber to *L. variegatus*. The experimental design was able to discern differences between the growth in the two sediments, although it did not find effect caused by microrubber. This supports the use of *L. variegatus* as a model animal to test the effects of microplastic particles in sediment. Lake Höytiäinen sediment has been used in several studies as substrate and considered as clean (Leppänen and Kukkonen 1998); it even is the substrate of our *C. riparius* cultures. On the other hand, the sediment from Huruslahti bay received historically a high load of chemicals from, for example, the wood processing industry and has a high load of tributyltin (TBT) and metals (Sahoo and Oikari 2016). Differences in the organic carbon (OC) are most probable cause of the differences found between the two sediments, most remarkable in the dry weight of the worms (Fig. 3). The OC content from lake Höytiänen sediments is approximately 3.5 times lower than the OC content in Huruslahti sediment (34.6 ± 0.13 and 125.43 ± 0.47 g kg sediment dry wt^−1^, respectively).

Articles that evaluate the mixture toxicity of microplastics and other environmental contaminants are scarce and almost absent for microrubber (Dolar et al. 2021). The mixture toxicity of chlorpyrifos and a similar microrubber produced distinct toxicity responses compared to chlorpyrifos alone in the terrestrial isopod *Porcellio scaber* (Dolar et al. 2021). However, some of these responses could be attributed to the sorption of the chemical to the microplastics, reducing its toxicity.

The only potential point where some effects could occur is in the reproduction. We found that in the fine-sized microrubber at 10% nominal concentration, *C. riparius* produced more egg ropes per female than in the rest of the treatments (Table 1). However, the significance of these results cannot be evaluated, as we only had one reproduction cage for each treatment due to the grouping of adults to maximize the production of egg ropes. To date, only negative or negligible effects of tire rubber on the reproduction of aquatic animals have been found (Panko et al. 2013; Halle et al. 2021) and therefore we recognize the need of future studies that corroborate them.

## Conclusions

Our results suggested a minimal effect of microrubber on the aquatic sediment dwellers *L. variegatus* and *C. riparius* in laboratory experiments at environmentally relevant concentrations and with different particle sizes and types of sediments. However, the present study does not necessarily reflect what occurs in the real environment, where the presence of tire rubber particles together with variations in environmental factors such as temperature, salinity and pH may negatively influence the wellbeing of sediment wildlife.

## Supplementary Information

Below is the link to the electronic supplementary material.

Supplementary file1 (DOCX 3478 kb)
