# Supplementary material for: Toxicity of Tire Rubber Microplastics to Freshwater Sediment Organisms
Source: Arch Environ Contam Toxicol. 2021 Dec 20;82(2):180–90. doi: 10.1007/s00244-021-00905-4 (PMC8818004; doi:10.1007/s00244-021-00905-4)
Supplement: Supplementary file 1 — Supplementary file1 (DOCX 3478 kb) [file 244_2021_905_MOESM1_ESM.docx]

Supplemental information

Toxicity of tire rubber microplastics to freshwater sediment organisms

Authors: Victor Carrasco-Navarro*, Aino Nuutinen, Jouni Sorvari and Jussi V. K. Kukkonen

Affiliation:

Department of Environmental and Biological Sciences, University of Eastern Finland,

Kuopio campus, PO Box 1627, FI-70211 Kuopio, Finland

*Corresponding author:

E-mail address: victor.carrasco.navarro@uef.fi (V. Carrasco Navarro)


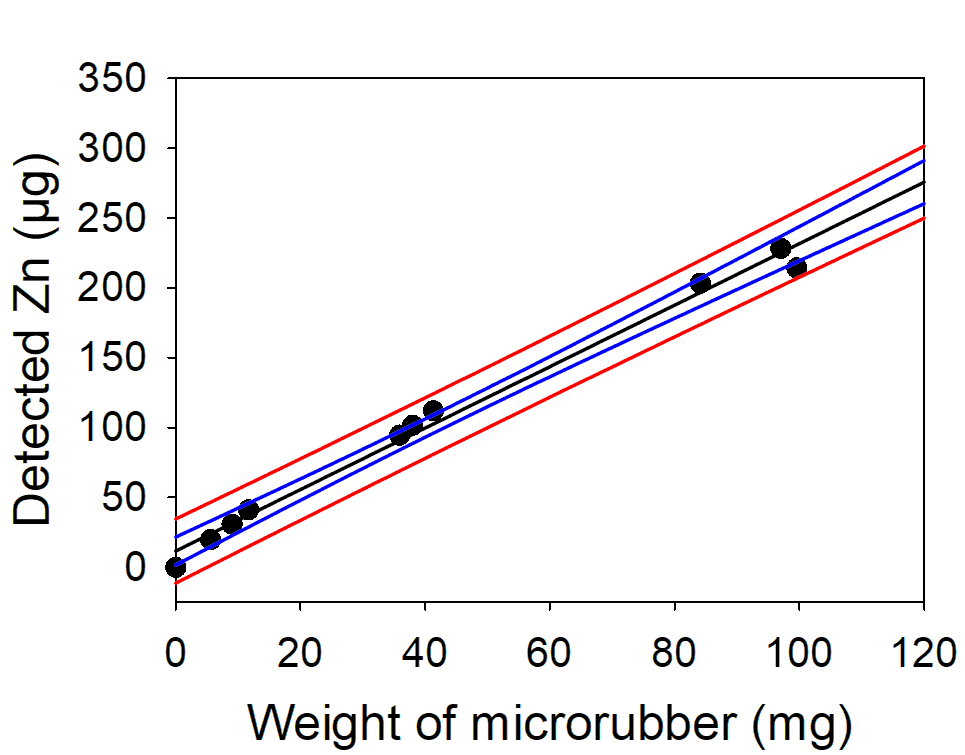


Fig. S1. Linear regression of the obtained Zn quantities (µg; y axis) with the amount of microrubber extracted (mg; x axis) with the acetic acid. The dots are the experimental values (n=9), that were corrected for a blank. The origin was included in the regression. Blue lines represent the 95% confidence bands and the red lines the 95% prediction bands. R^2^ = 0.9901; y0 = 11.76 (4.33), a= 2.2 (0.08).


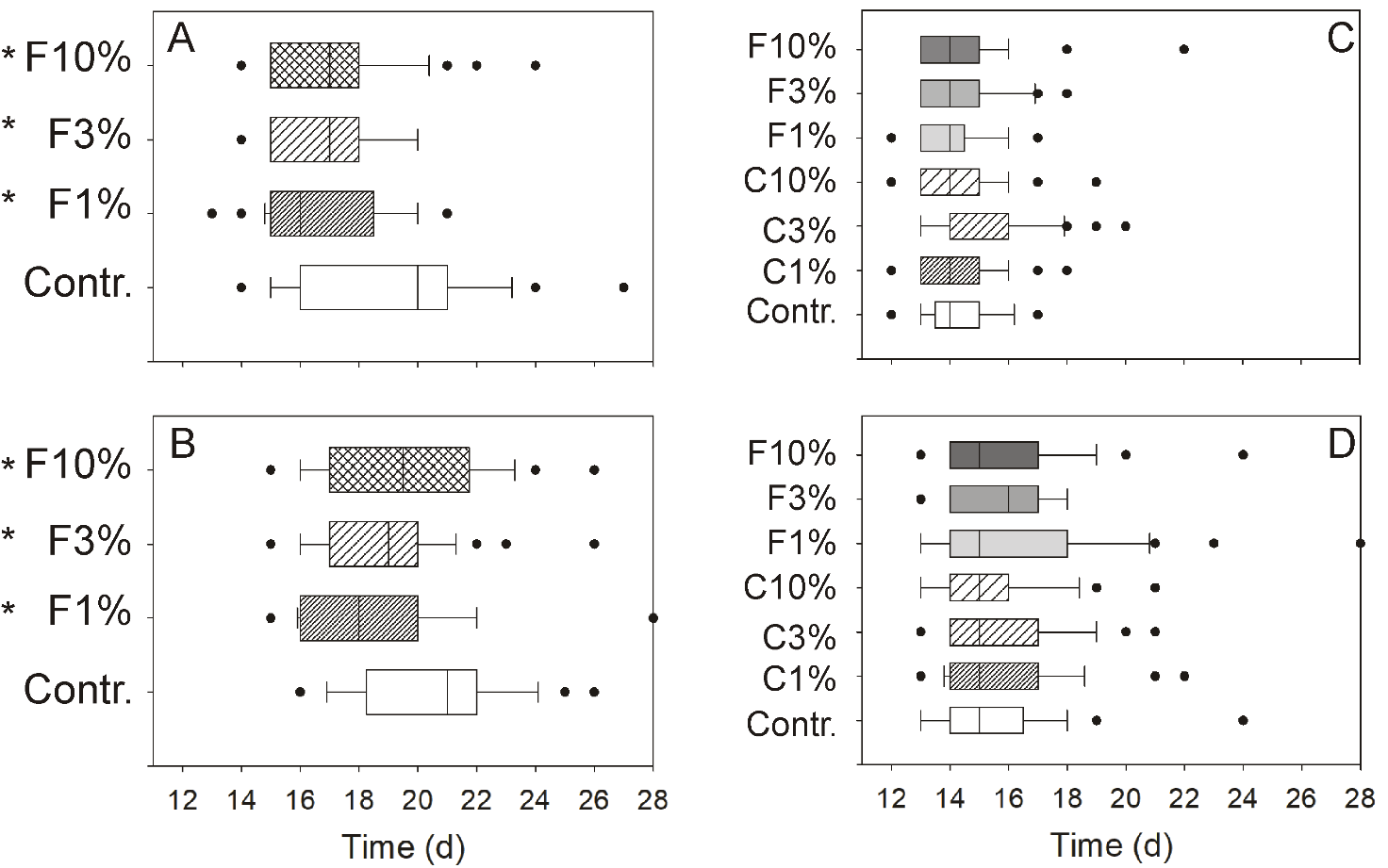


Fig S2. Emergence profiles of males and females in the Ruosmanlampi (A and B) and Höytiäinen sediments (C and D). F and C indicate the fine and coarse sized microrubber, respectively. The nominal concentrations are indicated after the F or C (1 to 10%). Contr. indicates controls. Asterisks denote statistical significance regarding to corresponding controls.


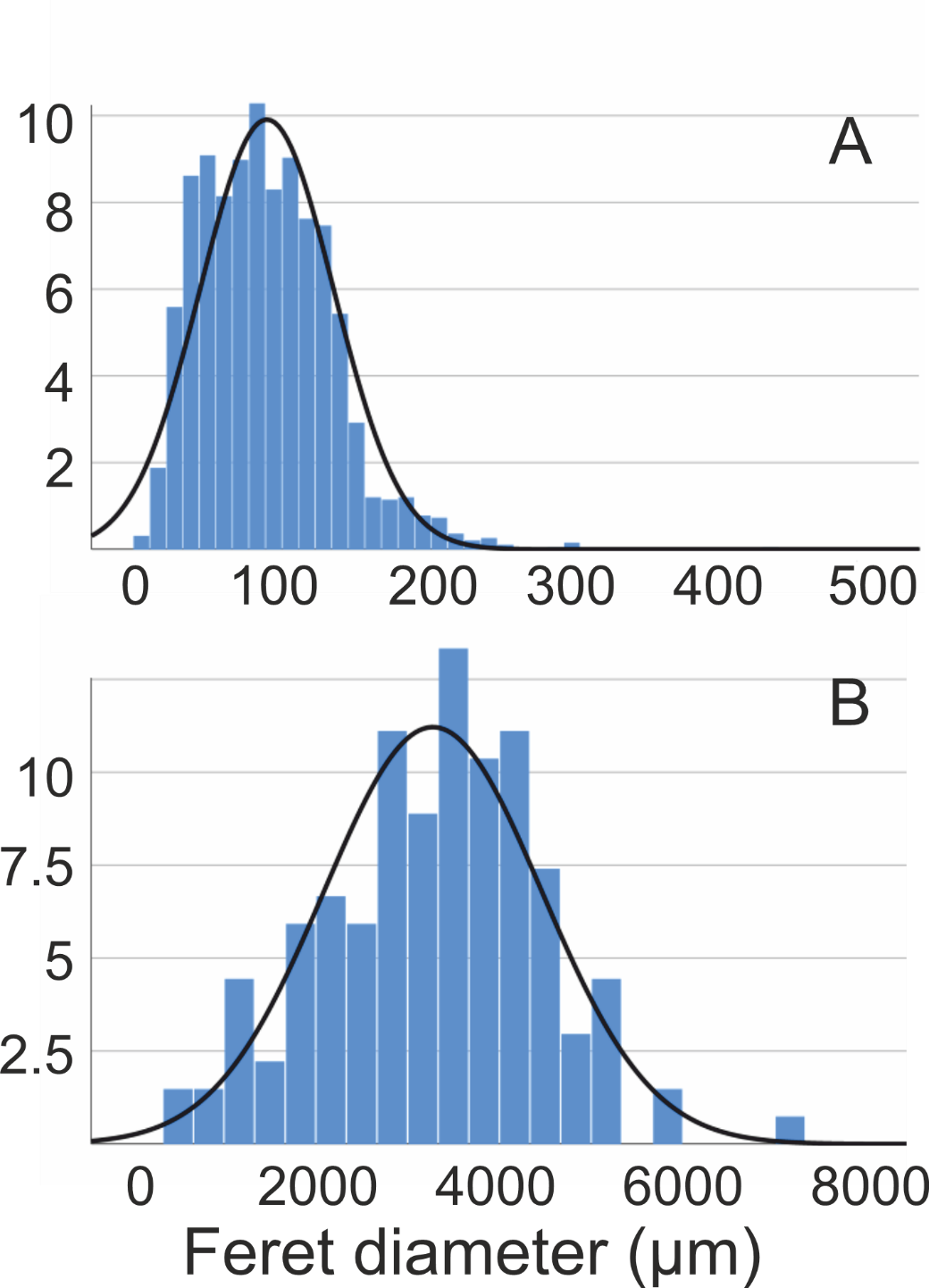


Fig. S3. Particle size distribution curves for the fine particle size (A) and the coarse particle size (B).


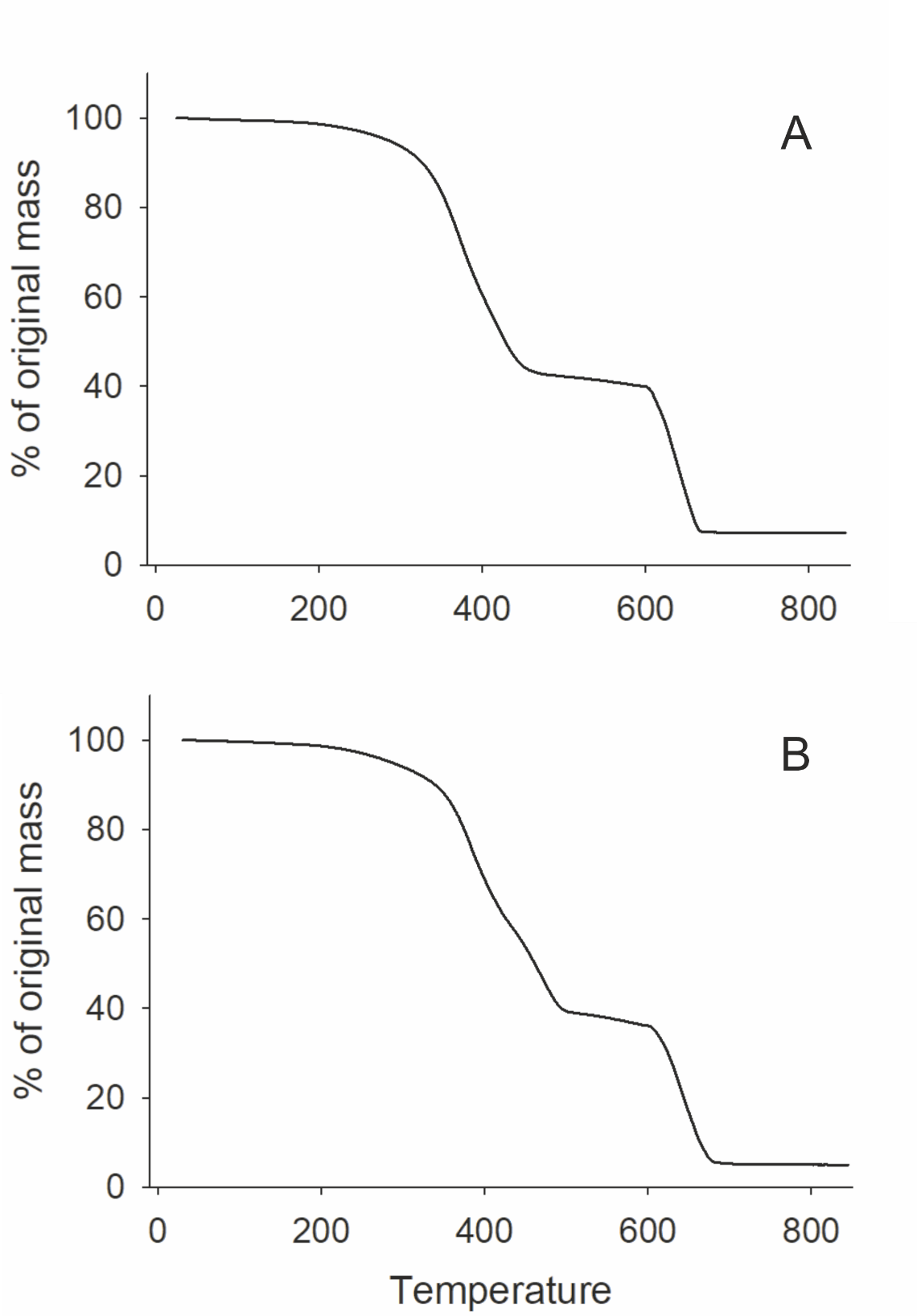


Fig. S4. Decrease in the weight of the fine (A) and the coarse (B) size microrubber during thermogravimetric analyses (TGA).

Table S1. Thermogravimetric analyses (TGA) profile of the fine and coarse sizes used in this study. The percentages are the weight loss during the showed increase in temperature, indicating the composition of the materials used.

| **Microrubber components** | **Temperature range** | **Fine size** | **Coarse size** |
| --- | --- | --- | --- |
| Volatile compounds | 30-300 °C | 6.28% | 5.97% |
| Polymers | 300-600 °C | 53.8% | 57.9% |
| Carbon black | 600-850 °C | 32.66% | 31.10% |
| Remaining residue, inorganic fillers | - | 7.3% | 5.01% |
